# Physical Activity in the Daily Life of Adolescents: Factors Affecting Healthy Choices from a Discrete Choice Experiment

**DOI:** 10.3390/ijerph17186860

**Published:** 2020-09-19

**Authors:** Sabina De Rosis, Ilaria Corazza, Francesca Pennucci

**Affiliations:** Management and Healthcare Laboratory, Institute of Management and Department EMbeDS, Scuola Superiore Sant’Anna, 56127 Pisa, Italy; i.corazza@santannapisa.it (I.C.); f.pennucci@santannapisa.it (F.P.)

**Keywords:** lifestyle, healthy habits, active life, adolescents, physical activity, daily travel choice, discrete choice experiment

## Abstract

Physical activity improves peoples’ well-being and can help in preventing weight gain, obesity, and related non-communicable diseases. Promoting healthy behaviors in the daily travels and transport choices of adolescents is very important in early establishing healthy habits that imply routine physical activity. For designing and developing effective strategies, it is relevant to study adolescents’ preferences for physical activity and what factors in the social and environmental contexts affect their preferences. The paper investigates these aspects by means of a discrete choice experiment, using data from more than 4300 16–17 year-old adolescents in Italy. The results show that adolescents generally prefer walking for long time alone. However, females prefer cycling, while adolescents from lower educated families prefer motorized means of transport. Environmental factors affect the adolescents’ preferences: living nearby a green area is associated with more active and healthier choices in their short daily travels. Conversely, adolescents living closer to an industrial or high traffic area prefer to use motorized vehicles. Such findings have been discussed and policy implications presented, in order to support policymakers in designing cross-sectoral policies to promote healthy choices related to physical activity in adolescence.

## 1. Introduction

The epidemic dimensions of obesity, the diffused onset of chronic diseases, and the consequent sustainability of social and health spending in Western countries are very critical issues [1,2,3]. More than two thirds of premature adult deaths are linked to non-communicable diseases (NCDs), reflecting behaviors started or reinforced during adolescence [4,5]. A healthy lifestyle can prevent inactivity and weight gain in the younger population [2,6,7,8,9]. On the contrary, suboptimal physical activity (PA) is an obesity risk factor during childhood and adolescence, and is increasing all over Europe [10]. In this respect, both positive and negative behaviors developed during adolescence are taken forward into adult life [11,12], also leading to eating disorders, obesity, associated morbidity and, more generally, chronic NCDs in adulthood [4,13,14,15]. In NCD-predominant countries, as many as one adolescent in every three is obese [16], and there has been an increase in physical inactivity during adolescence [17,18,19]. Globally, the most of young people do not practice sufficient PA to achieve health benefits [20,21,22]. Fewer than one in four adolescents meet recommendations for PA (i.e., 60 min per day) [16].

The prevention of obesity can be effectively addressed in an early period of life. Indeed, health promotion at a young age is closely linked to the current and future sustainability of the health systems [3,4,23,24]. Targeting health promotion policies to this specific age group is necessary to establish healthy behaviors early in the lifespan of individuals, thereby positively affecting future behaviors [25]. In this regard, on the one hand, younger people, and in particular adolescents, the so-called post-millennials or Z generation, are in a key phase of their life, when behaviors can change and new habits can be established. On the other hand, adolescents make autonomous daily decisions in several aspects that can impact their level of PA, such as short trips like the home–school travel or dates with friends. In addition, they are particularly interested in the environmentally friendly domain [26,27,28], that can push them in choosing an alternative mean of transport that is healthy and sustainable (i.e., walking instead of motorized transport).

Promoting PA in adolescence has been found to be effective in lowering the risk of NCDs in adulthood [29,30,31,32,33]. Among the various strategies, several government bodies and professionals have addressed the challenges related to obesity by promoting PA and reducing sedentary behaviors. As mentioned above, several recommendations are available, indicating a recommended level of PA for young people [34,35,36]. Several NCD countries have implemented promotion interventions [37,38], and adopted strategies targeted to adolescents, including active travel to school and after school activities [37], with the aim of improving the level of PA during adolescence. In general, healthy choices related to transport and short travel can promote more active and healthy lifestyle of people, and increase their quality of life [39,40]. The focus on short travel presents great potential. However, the effectiveness of young people independent mobility, for instance by walking or cycling to school and other local places, was impaired by some environmental factors, such as the traffic congestion [37], poor public planning for pedestrians and cyclists, parental worries for safety [41,42], and risk of injury [43,44]. Active transport needs the engagement of local governments and communities, in order to plan, realize, manage, and promote the use of routes, places, and facilities [37]. Acting on the “environmental factors” affecting the PA may change the choice architecture of adolescents [38], and facilitate the increase of PA level in those communities where the urban design is PA supportive [45]. “Environmental variables” are defined as anything outside the individual that can affect his/her PA behavior [46].

Policymakers and scholars’ interest in expanding the knowledge on the factors affecting PA is high. This includes the need of a better understanding of the role that different factors at individual, social and environmental levels can have on changes in PA and sedentary behaviors [47,48]. Policymakers are interested in pushing or maintaining the adoption of healthy lifestyles by the population. Moreover, the recently renewed resonance of the climate change issue has increased the environmental concerns and motivated the spreading of sustainable behaviors, which include environmental-friendly lifestyle habits [49] or a “green lifestyle” [50]. The “green lifestyle” translates into actions that are pro-environmental and sustainable behaviors, including green consumption and transport, for instance, walking or cycling for short journeys instead of using a motor vehicle [50]. More recently, the COVID-19 pandemic has additionally emphasized the need to address physical inactivity among people, including children and young individuals who, in this unprecedented time, risk much more weight gain, obesity, and associated NCDs in adulthood [13,14].

Individuals still fail to make sustainable and healthy choices in everyday life [51,52,53,54]. In this respect, initiatives aimed at changing behaviors have increasingly adopted the ‘nudge’ approach of Thaler and Sunstein, by changing the choice architecture of individuals for presenting the ‘right’ choices as more appealing than the ‘wrong’ ones [55,56]. By following a less paternalistic approach, behavioral changing interventions should address not only individual cognitive biases, but also cultural and social contexts and dynamics, as illustrated by Pennucci and colleagues [57]. To this end, policymakers need information on population preferences and individual, social, and environmental factors that can affect the individual behaviors in relation to their healthy choices, as mentioned above.

### Factors Affecting the Level of PA in Adolescence

Despite the fact that research into determinants of PA behavior has recently increased, there is a scarce evidence on factors affecting the PA level specifically in the adolescence [45,58]. With regards to the determinants of PA among adolescents, positive associations with PA were found for:

(i) personal factors such as individual characteristics, sex (male), level of parental education; psychological factors such as self-acceptance or ‘appearance’, attitude, self-efficacy, goal orientation/motivation [59,60];
(ii) the typology of PA, for instance, leisure [61] or ‘enjoyment’ PA [58], technology-supported PA [62];
(iii) social/environmental factors, such as having co-participants (sub-groups of peers) practicing PA [60,63], also at school [62,64]; school type [46]; physical education/school sports, family influences [60]; built environment (i.e., neighborhood characteristics; presence of public open spaces; street/pedestrian connectivity/amenities; recreation land use proximity; parks/public open space install or improvements; traffic-related areas) [65,66];
(iv) PA timing and location [63], and costs and resources [62].

Previous research has studied lifestyle habits referring to PA meant specifically as transport choices for short travels. The transport decision-making of individuals is also affected by individual characteristics and psychological factors [67], related to emotional states, subjective preferences, motivations, identities, and perceptions [68]. In addition, price and convenience, environmental benefits, as well as social context, interactions, and norms have been identified as factors influencing transport-related behaviors [69,70,71,72,73,74,75]. Nevertheless, research on this specific domain is mainly focused on the adult population, while relatively little evidence is available on factors motivating adolescents to be physically active in their short travels [58,76]. Different factors can affect the behavior of people in different age groups [77]. In addition to the various psychological factors affecting the behavior of young people [78,79], peers, family, and social media can also influence the green and healthy behaviors of adolescents [75,78,80,81]. Urban design, and in particular the presence of infrastructure (e.g., pedestrian roads) and the communication of their availability, have been found as determinants of the adolescents’ choice architecture in terms of transport for short travels, as mentioned above [82,83].

Research that includes discrete choice modelling is aimed at capturing the effect of various of the above mentioned variables in studying the main preferences in short trips of the adult population, while little attention has been paid to younger people [75]. Identifying determinants of adolescents’ healthy choices of transport can inform policymakers to define strategies and campaigns for pushing healthy, and at the same time environmentally friendly, behaviors in the post-millennials’ mobility [58]. The present study is aimed at investigating what drives an adolescent in choosing healthy alternatives in his/her short travels. 

Therefore, the objectives of this study are: (1) to describe the profile, meant as their individual characteristics, of the adolescents who prefer healthy transport alternatives, (2) to identify social/environmental factors affecting the healthy choices of adolescents in relation to short travels, and (3) to provide suggestions to practitioners for a better understanding of this specific behavior of the Z generation and to inform future PA and transport-related policies.

## 2. Methodology

This study investigates adolescents’ preferences by means of a discrete choice experiment (DCE). Discrete choice models represent a class of experiments widely applied in the fields of marketing, transport, as well as in environmental, labor and health economics and policy [84,85,86,87,88]. They are ultimately intended to extrapolate respondents’ preferences starting from their stated preferences in hypothetical scenarios [89]. The authors selected such a kind of experiment with the aim to identify adolescents’ preferences for PA so as to allow the design of effectively tailored health promotion policies, because discrete choice models are considered as one of the most adequate techniques for preferences elicitation [90,91].

The data used in this research refer to a survey undertaken in the Tuscany Region (Italy) from November 2016 until March 2017, within the beFood initiative [92,93]. The theoretical sample size was estimated in 3572 respondents, stratified in the 10 provinces of Tuscany. More in details, the theoretical convenience reference sample was obtained for each province, starting from data published by the Italian National Institute of Statistics (ISTAT) as of 1 January 2016 referring to adolescents of age between 16 and 17 years for each of the Tuscan provinces (N = 62,177) [94], considering a level of significance *p* = 0.05 and tolerating a margin of error d = 0.05. Participants in the survey were not randomly selected, but joined the project via a snowball sampling procedure.

No ethical approval was needed for this study, as the informed consent was asked to adolescents’ parents at the time of first access to the online questionnaire. The beFood survey collected several data on lifestyle habits and preferences from more than 4700 16–17-year-old individuals (for ease of presentation, we will refer to these individuals as adolescents). The survey consists of a digital questionnaire administered using a web app. To characterize respondents, we used the following variables: sex, the highest level of parents/family education, body mass index (BMI), adherence to food and PA recommendations, being member of a sports association, being physically active (at least 60 min a day). The questionnaire also asked for information regarding the living environment of adolescents: industrial areas, high traffic streets, parks, pedestrian areas, country side (see Appendix A). In order to classify living areas by size (i.e., large vs medium-sized cities), the authors referred to specific methodologies of classification [95,96] and provided to respondents some examples in the options of answer. The adherence to food and PA recommendations from World Health Organization (WHO) and Tuscany Region [97] was computed as a composite index. In particular, the authors built two scores of adherence to the regional and international recommendations for healthy lifestyle behaviors, using the data on daily habits about nutrition and PA reported by the adolescents in the questionnaire. The two adherence scores are calculated for each respondent according to the frequency of consumption of each specific food product (food score—FS) and the typology and frequency of PA (physical activity score—PAS). The two scores present a minimum value that indicates the least adherence to the recommendations and a maximum that means the highest adherence to the recommendations. The FS measures the adherence of food habits to the healthy diet recommendations of Tuscany Region’s food pyramid and the official WHO recommendation on fruits/vegetables consumption 5 times a day [97,98]. The FS ranges from a minimum of −6 to a maximum of 27.5. The PAS measures the adherence of behaviors to the “60 min a day” WHO recommendation [99] and to the guidelines of Tuscany Region on PA [97]. The PAS ranges from a minimum of −1 to a maximum of 25. 

The questionnaire included a DCE [100] in order to obtain the elicitation of adolescents’ preferences about PA. In the experimental design phase, the adolescents were provided with a scenario to contextualize their choice: You are going out in the late afternoon. If you could choose, how would you move?. This scenario, pertaining to frequent short travel that can imply the choice of PA, was chosen because it is related to a potentially real-life situation where adolescents might act in making a decision. The adolescents were asked to choose between different alternatives based on attributes and levels, and characterizing the various choice sets [101]. The attributes adopted and the respective levels are summarized in Table 1 and were chosen on the basis of a previous literature analysis, as described in the introduction.

The cost of transport was excluded, because it could be more salient for parents or family, rather than for the individual adolescent. The distance was considered overlapping with the travel time and, therefore, it was not included among the attributes.

The full factorial experimental design, realized with the STATA14 software, produced 3^3^ (27) combinations, randomized to form 18 pairs (or choice sets), collected in 6 blocks of 3 pairs of alternatives [87]. Each participant responded to only one of the six blocks of the experimental design, as randomly selected. Every block was made of three choice sets containing, respectively, two alternatives each. The DCE was conducted at once. The participants viewed and were asked to respond the three choice sets sequentially. They could give only one answer and could not go back or change answers once provided. Each alternative was constructed using varying levels of the same attributes. The design of alternatives was developed in order to provide orthogonality, balance, and a minimum overlap across levels [102,103]. Table 2 shows an example of choice set presented to respondents:

For a full view of the questions related to PA behavior and of the blocks, choice sets and alternatives of the DCE, see respectively Table A1 in Appendix A and Table A2 in Appendix B.

The questionnaire reached 5029 individuals, of whom 4749 were 16–17 years old, of whom 4358 were suitable for data analysis since they completed the questionnaire. The DCE data were coded as dummy variables and analyzed through conditional logit modelling in STATA14 [104]. Respondent preferences were identified with respect to main effects and, secondly, interaction effects considering the characteristics of respondents (i.e., sex, BMI, higher level of education in the family and level of adherence of the diet of the respondent to the Tuscan food pyramid). Another conditional logit model was performed considering only factors related to where they live, operationalized as city size and type of neighborhood meant as proximity to one of the following: industrial areas, high traffic streets, parks, pedestrian areas, country side [66].

Finally, we performed a full conditional logit model including all above mentioned variables, with the exclusion of those that we found collinear, in order to observe the interactions between the preferences of the respondents and both their characteristics and the environmental factors.

## 3. Results

The characteristics of the group of adolescents participating in the DCE were analyzed (Table A3—Appendix C). In particular, more than a half (57%) were female, from a high (50%) or medium (39%) educated family. The average adolescents’ score of adherence to food and PA was calculated and it emerged that respondents’ lifestyle habits are not still fully adherent to the recommendations, with only 10% of participants having a high adherence profile. In relation to the living environment, the most of adolescents do not live in large or very large cities (8.1%), with almost a half of them living in a village or town (48.5%). While the majority of adolescents participating to the DCE reported to live nearby an open or green place (44.5% live near a park, 21.5% near a pedestrian zone, 34.9% near the countryside), around a quarter of them reported living near a busy road, and a small percentage (5.5%) near an industrial area.

The analysis of main effects from the collected data showed that, in general, the less preferred mean of transport by adolescents is the bike; they prefer longer travels in terms of time and to move alone (*p* < 0.001; Table A4—Appendix C).

Considering the socio-demographic factors and the personal characteristics of adolescents, it emerged that females’ most preferred choice is the bike (*p* < 0.001). Motorized vehicles are the preferred by adolescents who come from a family with a medium level of education (*p* = 0.005). This group prefers to move alone rather than with parents (*p* = 0.009). Similarly, adolescents who prefer to be members of sport associations prefer to move alone with motorized means of transport (see Table A5—Appendix C). On the contrary, adolescents who report to practice PA for almost 60 min per day prefer walking rather than using motorized means of transport (*p* < 0.001), but for not too long (*p* = 0.02). Finally, it is worth pointing out that the lower the adherence to food and PA guidelines, the higher the preference for choosing the bike (Table A5—Appendix C). 

By analyzing the results of the models, including only the environmental/neighborhood factors (Table A6—Appendix C), it emerges that adolescents always prefer to move alone as compared with going with friends or with family, independently from the characteristics of the place where they live (*p* < 0.005). Similarly, 45 min is the preferred travel time in any case (*p* < 0.001), although for living in a metropolitan area and nearby an industrial area, it is not statistically significant.

The preferred way of moving is walking when adolescents live in medium-low urbanized places (*p* < 0.001). However, they are also likely to use motor transport if they live in villages (*p* < 0.05). The neighborhood configuration is a key factor: living close to a park or a pedestrian area is associated with a preference for walking, while living not far from the countryside is associated with preferring both walking and using a motorized vehicle (*p* < 0.001). The bicycle is not a favorite choice for adolescents living nearby a high traffic or industrial area (respectively, *p* < 0.001 and *p* = 0.038).

Table A7 (Appendix C) presents the result of the full conditional logit model, where all variables have been computed. The results that remained statistically significant confirm that, while adolescents in general prefer walking, female adolescents prefer moving by bike (*p* < 0.001). The results from the full model confirm that the lower the adherence to the food and PA guidelines, the higher the preference of adolescents towards using motorized means of transport (*p* < 0.001). Being member of a sport association is related with using bike (*p* = 0.001) and motor vehicles (*p* = 0.002); while being active with almost 60 min of PA per day, living nearby a park or a pedestrian area is associated with choosing a healthy mean of transport (respectively, *p* < 0.001, *p* = 0.003; *p* = 0.017). It is also confirmed that active adolescents prefer shorter travel time (*p* = 0.02). In relation to the effect of co-participants in practicing PA (walking and biking) for daily travels, the full model shows that more active adolescents prefer to move with parents (*p* = 0.025), while adolescents living nearby an industrial area prefer not to move with peers (*p* = 0.007).

## 4. Discussion

This paper examined the preferences of adolescents among alternatives for their daily short travel choices, by describing the profile of adolescents who prefer healthy means of transport and the social/environmental factors associated with the tendency to prefer healthy short means of transport.

Sex is revealed as an influencing factor for the preferences of typology of PA, with females preferring cycling to walking and to using motorized means of transport. According to previous research, youths and adolescents can achieve a better health by cycling more than by walking [105,106]. To this respect, the results here presented suggest that implementing interventions to promote cycling should be useful for improving the healthiness of adolescents and especially for males and those who come from lower educated families. In fact, among developed countries, those with long traditions and comprehensive infrastructures related to cycling, have higher rates of cycling versus walking to school among adolescents [107].

The results showed that adolescents who have already adopted healthy habits, such as practicing almost 60 min a day of PA, or following food and PA guidelines, are more likely to prefer walking for their short travels. Choosing healthy and green alternatives for moving, such as walking or cycling, can be considered a routinely repeated behavior in daily life of this group of adolescents. Nevertheless, previous research has highlighted that, for specific groups of population (i.e., elderly), being physically active and educated on healthy food behaviors are not sufficient conditions to follow PA recommendations in everyday life [108].

As proposed by De Rosis et al. [78], when designing health promotion initiatives following the peer-to-peer approach, the groups of adolescents characterized by healthy behaviors could be trained, activated, and engaged as positive testimonials and facilitators of positive lifestyles towards their peers, thus promoting healthier communities. Nevertheless, peer-to-peer initiatives are not a priori successful [109,110,111,112]. To this respect, it is worth pointing out that the findings of this DCE clearly highlight that, in general, adolescents do not prefer alternatives of travel that encompass friends, neither parents. In a previous study, the lack of friends was reported by adolescents as a barrier to PA [113], while the presence and social support of friends and family was found as positively associated with the PA level of adolescents [114,115]. This was found also in a study related to the active transport choices of adolescents [116]. Social factors can influence adolescents’ choices, driving them towards either a more or a less healthy/active transport alternative [117]. The elicited preferences for being alone in their short travel can be explained by the willingness to be autonomous in their choices of transport mode [118].

At the same time, family can be a protective and positive agent of influence on adolescents. Our results emphasize that adolescents from more educated families tend to prefer healthier alternatives of travel. Previous studies have demonstrated that parents and, more in general, relatives can be positive influencers [119,120]. Nevertheless, according to these study findings, adolescents prefer to practice PA for their daily travels without parents or family. Thus, targeting family and peers in promoting active lifestyles should be considered with caution. The actual evidence suggests that social support positively affects the PA, although the association is still unclear and supported by mixed evidence [121,122,123,124,125]. Community interventions should take into consideration the whole and complex choice architecture of individuals, considering both the individual’s family and the specific cultural and social contexts and dynamics [57,126].

In addition, it emerged that being member of sport associations does not contribute in the establishment of good daily travel habits. It is arguable that this group of adolescents prefer to be active practicing sport rather than move for reaching their leisure destinations, or that they think that their sport activity is enough and that they do not need additional PA. However, further research should be conducted on these hypotheses and more in general on how sporty and less sporty adolescents move in their routine life [126,127]. The implication of this result can be a rethinking of the role of sport association in promoting and facilitating healthy habits in the daily life of adolescents, by trying to increase the level of involvement and engagement of these associative actors in initiatives aimed at promoting a healthy lifestyle. Some experiences in other countries showed positive effects in this direction [128].

The study highlighted the importance of cross-sectoral policies aimed at promoting PA in the daily life of adolescent, including policy areas such as education, environment and transport. In fact, the proximity to green and pedestrian zones in urban areas is associated with a higher preference for healthy means of transport such as cycling and walking [129,130,131]. Previous research involving university students provided mixed evidence on the association between the perceived greenness of the environment and PA, with a negative association for sedentariness and perceived greenness at home, but not at university [132]. This confirms that it is key to know the adolescents’ preferences, in addition to the general information available on actual PA and main mode of transport used for the daily activities in adolescence. Once the preferences are clear, policymakers can plan actions to change the environmental choice architecture of adolescents and increase the opportunities of active travel, leisure activities involving PA and, as a consequence, increase their well-being. Considering the findings of this study, planning cycling and walking infrastructures regardless of the current used means of transport can be identified as an appropriate and concrete action for the delivery of PA and health-related strategies or policies [39,133].

In this respect, it can be observed that living in a village, nearby the countryside, or close to an industrial area is associated with a preference for motorized means of transport. This can be explained by the need of adolescents to reach distant areas. Furthermore, parents are sometimes not comfortable in allowing adolescents to walk or cycle in these contexts because of the perceived danger especially when looking at more deprived areas [126,134]. In this case, policies and incentives for promoting active travel (e.g., walking, cycling) should also be defined at local and regional levels, considering both temporal and spatial dimensions [132,133]. 

The differences across adolescents’ groups (males/females, from high/low educated families, living nearby green areas or not) reflect differences in PA opportunities and preferences, as well as to the value given to the PA itself. More vulnerable adolescents coming from disadvantaged contexts should be carefully targeted by specific incentives and policies [78,134,135].

### Limitations Strength and Future Research

The presented analysis is a further step in highlighting which factors influence the transport preferences of adolescents that imply PA.

Some limitations of this study are related to the self-reported questionnaire that can imply a misinterpretation of the questions, as well as social desirability in selecting the answers and decreasing attention, particularly in filling-in a DCE. Some of these potential biases have been mitigated by a great number of respondents that represent a very large sample for a robust experiment of discrete choice.

Another aspect of strength is represented by the replicability of the study. To this regard, future research could test applied interventions designed according to the results of this paper, in order to investigate whether adolescents actually make healthier and more active choices in their short travels, by addressing the factors affecting their elicited preferences.

Another further development could be related to investigate how and how much family and peers’ actual habits have an influence on adolescents’ choices in terms of transport. Some of these influences could then be used to frame adolescents’ choices and orient them to make better decisions. Similar research can be conducted also on different age groups, as well as in different cultural and geographical contexts. In fact, the more the ‘taylorization’ of the interventions, the better the results in terms of influencing behaviors. Lastly, a comparison between intentions and actual behaviors of the same respondents would be useful in studying how the attitudes relate to choices in daily routine contexts.

## 5. Conclusions

The results of the presented DCE in this study confirm that the environmental choice architecture is a key aspect in determining the preferences of adolescents, with a positive association between the proximity to green areas and walking or cycling. The study also suggests that the family has an influence on the active and healthy choices of adolescents in their short travels, but that they generally prefer to be alone when the means of transport are their legs or bikes.

Policies aimed at supporting adolescents in making healthy PA choices should take into consideration these findings when designing community and cross-sectoral interventions.

## Figures and Tables

**Table 1:** Attributes and levels of the DCE.

Attributes	Levels
Mean of transport [66]	By foot
	By bike
	Motor transport (i.e., bus, car)
Travel time [63,66]	15 min
	30 min
	45 min
Type of company [62,63,64]	Alone
	With friends
	With parents

**Table 2:** Example of a choice set.

SCENARIO: Imagine You Are Going Out in the Late Afternoon… If You Could Choose, How Would You Move?
Alternative A: I would move by bike for 30 min with friends
Alternative B: I would move by foot for 45 min alone

## Appendix A. Questionnaire

**Table A1:** Questions used as variables for the analysis of the DCE results.

Questions	Options of Answer
You are …	Male/Female
What is the highest educational qualification in your household, excluding yours?	I don’t know None Primary school Middle school High school Bachelor Master and/or Ph.D.
Are you a member of a sports association?	Yes/No
Do you eat fresh or cooked fruit or vegetables (not including potatoes)?	Everyday 5–6 times per week 3-4 times per week 1-2 times per week Less than once per week Never
Do you eat pasta, bread, rice or spelt?	
Do you eat salty snacks such as chips or nuts?	
Do you eat beans like as green peas, lentils, or soy?	
Do you eat fish?	
Do you eat white meat like as chicken or turkey meats?	
Do you eat eggs?	
Do you eat fresh or matured cheese?	
Do you eat red meat like as beef steak?	
Do you eat processed red meat such as burgers or salami?	
Do you eat sweets like as biscuits, ice cream, brioches or sugary snacks?	
Do you eat milk or yogurt?	
Do you eat pizza or piadina?	
Do you eat kebab?	
Do you drink beer or wine?	
Do you drink beverages like as tea, coke or fruit juice?	
Do you eat fresh-squeezed juice at home or in a bar?	
Did you do at least 60 min / day of moderate (e.g., walking) or vigorous (e.g., swimming) activity?	Yes/No
You live in:	A very large city, such as Rome or Milan A large city, such as Florence or Bologna A medium-sized city, such as Pisa or Ancona A town or a village
You live near a park…	Yes/No
You live near a pedestrian zone…	
You live near the countryside…	
You live near a busy road…	
You live near an industrial area…	

## Appendix B. DCE

**Table A2:** Blocks, choice sets and alternatives of the discrete choice experiment.

Block	Choice Set	Alternative	Mean of Transportation	Travel Time	Type of Company
1	1	1	By bike	30 min	With friends
1	1	2	By foot	45 min	Alone
1	2	1	By foot	30 min	With parents
1	2	2	By bike	15 min	With friends
1	3	1	Motor transport (i.e., bus, car)	15 min	Alone
1	3	2	Motor transport (i.e., bus, car)	45 min	With parents
2	4	1	Motor transport (i.e., bus, car)	30 min	With friends
2	4	2	By foot	45 min	With parents
2	5	1	By foot	15 min	Alone
2	5	2	Motor transport (i.e., bus, car)	45 min	With friends
2	6	1	By bike	15 min	With parents
2	6	2	Motor transport (i.e., bus, car)	30 min	Alone
3	7	1	Motor transport (i.e., bus, car)	45 min	Alone
3	7	2	By bike	30 min	With parents
3	8	1	By foot	45 min	With friends
3	8	2	Motor transport (i.e., bus, car)	15 min	With friends
3	9	1	By foot	30 min	Alone
3	9	2	By foot	15 min	With parents
4	10	1	Motor transport (i.e., bus, car)	15 min	With parents
4	10	2	By foot	45 min	Alone
4	11	1	By bike	30 min	Alone
4	11	2	By foot	15 min	With friends
4	12	1	By bike	30 min	With parents
4	12	2	Motor transport (i.e., bus, car)	30 min	With friends
5	13	1	By foot	30 min	With friends
5	13	2	By bike	15 min	Alone
5	14	1	By foot	15 min	Alone
5	14	2	Motor transport (i.e., bus, car)	45 min	With friends
5	15	1	By bike	15 min	With parents
5	15	2	Motor transport (i.e., bus, car)	30 min	With parents
6	16	1	Motor transport (i.e., bus, car)	15 min	With parents
6	16	2	By foot	45 min	With friends
6	17	1	Motor transport (i.e., bus, car)	45 min	Alone
6	17	2	By foot	30 min	With parents
6	18	1	By bike	30 min	Alone
6	18	2	By bike	15 min	With friends

## Appendix C. Results

**Table A3:** Characteristics of respondents.

Variables	Answers	%
Gender	Female	57%
BMI class	Underweight	18.2%
	Normal weight	71.3%
	Overweight	8.2%
	Obese	2.3%
Educational level	Middle school or High school	39%
	Bachelor, Master and/or Ph.D.	13.6%
Are you a member of a sports association?	Yes	54%
Did you do at least 60 min/day of moderate (e.g., walking) or vigorous (e.g., swimming) activity?	Yes	42.9%
You live in:	A very large city	0.5%
	A large city	7.6%
	A medium-sized city	43.4%
	A town or a village	48.5%
You live near a park …	Yes	44.5%
You live near a pedestrian zone …	Yes	21.5%
You live near the countryside …	Yes	34.9%
You live near a busy road …	Yes	24.2%
You live near an industrial area …	Yes	5.5%
Adherence to lifestyle recommendations	Low adherence	9%
	Medium adherence	81%
	High adherence	10%

**Table A4:** Analysis of main effects driving adolescents’ preferences.

Choice	Coef.	Std. Err.	z	*P* > |z|	[95% Conf. Interval]	
By bike	−0.5766	0.0359	−16.03	**0.000**	−0.6472	−0.5061
Motor transport	0.0255	0.0277	0.92	0.356	−0.0287	0.0799
30 min	0.3373	0.0305	11.03	**0.000**	0.2774	0.3972
45 min	0.9805	0.0307	31.91	**0.000**	0.9203	1.0407
With friends	−0.7560	0.0300	−25.16	**0.000**	−0.8149	−0.6971
With parents	−1.1326	0.0353	−32.00	**0.000**	−1.2020	−1.0633

Coef. = regression coefficients; Std. Err. = standard error; z = z value (test statistic z is the ratio of the Coef. to the Std. Err. of the respective predictor); *P* > |z| = probability that the z value would be observed under the null hypothesis that a particular predictor’s regression coefficient is zero, given that the rest of the predictors are in the model; 95% Conf. Interval = 95% confidence interval; the bold indicates statistical significant results.

**Table A5:** Analysis of interaction effects driving adolescents’ preferences: Adolescents’ socio-demographics and personal characteristics.

Variable	Coef.	Std. Err.	z	*P* > |z|	[95% Conf. Interval]	
Female * bike	0.3335	0.0757	4.40	**0.000**	0.1850	0.4819
Female * motor tr	0.0939	0.0586	1.60	0.110	−0.0211	0.2089
Female * 30 min	−0.0708	0.0651	−1.09	0.277	−0.1984	0.0568
Female * 45 min	−0.0657	0.0650	−1.01	0.313	−0.1932	0.0618
Female * friends	0.0499	0.0634	0.79	0.431	−0.0743	0.1743
Female * parents	−0.1349	0.0750	−1.80	0.072	−0.2821	0.0121
BMI * bike	2.77 × 10^−7^	1.12 × 10^−6^	0.25	0.804	−1.91 × 10^−6^	2.46 × 10^−6^
BMI * motor tr	1.04 × 10^−6^	6.25 × 10^−7^	1.66	0.096	−1.85 × 10^−7^	2.26 × 10^−6^
BMI * 30 min	9.52 × 10^−8^	6.83 × 10^−7^	0.14	0.889	−1.24 × 10^−6^	1.43 × 10^−6^
BMI * 45 min	−8.28 × 10^−7^	7.51 × 10^−7^	−1.10	0.270	−2.30 × 10^−6^	6.45 × 10^−7^
BMI * friends	2.69 × 10^−7^	6.66 × 10^−7^	0.40	0.686	−1.04 × 10^−6^	1.57 × 10^−6^
BMI * parents	7.03 × 10^−7^	7.66 × 10^−7^	0.92	0.359	−7.99 × 10^−7^	2.20 × 10^−6^
Low edu * bike	0.0462	0.1233	0.37	0.708	−0.1955	0.2880
Low edu * motor tr	0.1013	0.0950	1.07	0.286	−0.0849	0.2876
Low edu * 30 min	−0.1188	0.1037	−1.15	0.252	−0.3223	0.0845
Low edu * 45 min	−0.1638	0.1036	−1.58	0.114	−0.3670	0.0393
Low edu * friends	−0.1006	0.1032	−0.97	0.330	−0.3029	0.1016
Low edu * parents	−0.0126	0.1203	−0.11	0.916	−0.2485	0.2231
Medium edu * bike	0.0739	0.0789	0.94	0.349	−0.0807	0.2287
Medium edu * motor tr	0.1539	0.0606	2.54	**0.011**	0.0350	0.2728
Medium edu * 30 min	0.1229	0.0674	1.82	0.068	−0.0091	0.2551
Medium edu * 45 min	0.0810	0.0677	1.20	0.232	−0.0517	0.2138
Medium edu * friends	−0.0535	0.0657	−0.81	0.416	−0.1824	0.0753
Medium edu * parents	−0.1676	0.0779	−2.15	**0.032**	−0.3205	−0.0148
Low Adher * bike	−0.0681	0.1691	−0.40	0.687	−0.3996	0.2634
Low Adher * motor tr	0.5972	0.1305	4.57	**0.000**	0.3413	0.8530
Low Adher * 30 min	−0.2040	0.1448	−1.41	0.159	−0.4879	0.0799
Low Adher * 45 min	−0.1572	0.1437	−1.09	0.274	−0.4389	0.1244
Low Adher * friends	0.0996	0.1403	0.71	0.478	−0.1754	0.3748
Low Adher * parents	−0.0406	0.1661	−0.24	0.807	−0.3662	0.2849
Medium adher * bike	−0.0608	0.1208	−0.50	0.614	−0.2976	0.1759
Medium adher * motor tr	0.2451	0.0935	2.62	**0.009**	0.0617	0.4285
Medium adher * 30 min	0.0119	0.1026	0.12	0.907	−0.1892	0.2132
Medium adher * 45 min	0.0596	0.1021	0.58	0.559	−0.1405	0.2598
Medium adher * friends	−0.0593	0.1007	−0.59	0.556	−0.2568	0.1381
Medium adher * parents	−0.1838	0.1167	−1.57	0.115	−0.4127	0.0449
Assoc member * bike	0.2648	0.0781	3.39	**0.001**	0.1116	0.4179
Assoc member * motor tr	0.2031	0.0604	3.36	**0.001**	0.0845	0.3216
Assoc member * 30 min	0.0908	0.0665	1.36	0.172	−0.0395	0.2212
Assoc member * 45 min	0.0644	0.0668	0.96	0.335	−0.0664	0.1953
Assoc member * friends	−0.1533	0.0654	−2.34	0.019	−0.2817	−0.0250
Assoc member * parents	−0.1300	0.0769	−1.69	0.091	−0.2808	0.0206
60 min/day * bike	0.1111	0.0769	1.44	0.149	−0.0397	0.2620
60 min/day * motor tr	−0.2386	0.0601	−3.96	**0.000**	−0.3566	−0.1206
60 min/day * 30 min	0.0318	0.0662	0.48	0.630	−0.0980	0.1617
60 min/day * 45 min	−0.1553	0.0661	−2.35	**0.019**	−0.2849	−0.0257
60 min/day * friends	0.0704	0.0647	1.09	0.277	−0.0564	0.1973
60 min/day * parents	0.1653	0.0760	2.17	**0.030**	0.0162	0.3143

* indicates the interaction between two variables; Coef. = regression coefficients; Std. Err. = standard error; z = z value (test statistic z is the ratio of the Coef. to the Std. Err. of the respective predictor); P > |z| = probability that the z value would be observed under the null hypothesis that a particular predictor’s regression coefficient is zero, given that the rest of the predictors are in the model; 95% Conf. Interval = 95% confidence interval; the bold indicates statistical significant results; e- in the coefficients indicates an exponential coefficient.

**Table A6:** Analysis of interaction effects driving adolescents’ preferences: (a) city size/typology where adolescents live; (b) type of neighborhood.

Variable (a)	Coef.	Std. Err.	z	*P* > |z|	[95% Conf. Interval]	
Metropolis * bike	−0.7157	0.7699	−0.93	0.353	−2.224	0.7933
Metropolis * motor tr	−0.2518	0.4780	−0.53	0.598	−1.1887	0.6850
Metropolis * 30 min	−0.3750	0.5400	−0.69	0.487	−1.4334	0.6833
Metropolis * 45 min	0.4912	0.5788	0.85	0.396	−0.6432	1.6257
Metropolis * friends	−0.1167	0.5758	−2.03	**0.043**	−2.2959	−0.0387
Metropolis * parents	−1.6507	0.7154	−2.31	**0.021**	−3.053	−0.2484
City * bike	−0.5731	0.1297	−4.42	**0.000**	−0.8275	−0.3187
City * motor tr	−0.1595	0.1005	−1.59	0.113	−0.3566	0.0375
City * 30 min	0.3869	0.1083	3.57	**0.000**	0.1745	0.5993
City * 45 min	0.9753	0.1111	8.77	**0.000**	0.757	1.1932
City * friends	−0.6130	0.1077	−5.69	**0.000**	−0.8242	−0.4017
City * parents	−1.2308	0.1270	−9.69	**0.000**	−147.992	−0.9818
Town * bike	−0.5524	0.0542	−10.18	**0.000**	−0.6588	−0.4460
Town * motor tr	−0.0151	0.0420	−0.36	0.718	−0.0976	0.0672
Town * 30 min	0.2946	0.0463	6.35	**0.000**	0.2037	0.3856
Town * 45 min	0.9595	0.0465	20.61	**0.000**	0.8683	1.0508
Town * friends	−0.7502	0.0454	−16.49	**0.000**	−0.8393	−0.6610
Town * parents	−1.1055	0.0535	−20.65	**0.000**	−0.1210	−1.0006
Village * bike	−0.6001	0.0519	−11.55	**0.000**	−0.7018	−0.4983
Village * motor tr	0.0916	0.0399	2.29	**0.022**	0.0133	0.1699
Village * 30 min	0.3720	0.0441	8.43	**0.000**	0.2855	0.4584
Village * 45 min	1.0067	0.0442	22.74	**0.000**	0.9199	0.1093
Village * friends	−0.7812	0.0433	−18.03	**0.000**	−0.8661	−0.6963
Village * parents	−1.1376	0.0510	−22.29	**0.000**	−1.2377	−1.0376
**Variable (b)**	**Coef** **.**	**Std. Err.**	**z**	***P* > |z|**	**[95% Conf. Interval]**	
Park * bike	−0.4543	0.0596	−7.62	**0.000**	−0.5712	−0.3374
Park * motor tr	−0.1076	0.0462	−2.33	**0.020**	−0.1982	−0.0170
Park * 30 min	0.1762	0.0501	3.52	**0.000**	0.0780	0.2745
Park * 45 min	0.6004	0.0503	11.92	**0.000**	0.5016	0.6992
Park * friends	−0.4981	0.0497	−10.02	**0.000**	−0.5955	−0.4006
Park * parents	−0.7727	0.0581	−13.29	**0.000**	−0.8867	−0.6587
Pedastr * bike	−0.3440	0.0833	−4.13	**0.000**	−0.5073	−0.1807
Pedastr * motor tr	−0.1134	0.0644	−1.76	0.078	−0.2398	0.0128
Pedastr * 30 min	0.1389	0.0722	1.92	0.054	−0.0026	0.2806
Pedastr * 45 min	0.4798	0.0720	6.66	**0.000**	0.3387	0.6210
Pedastr * friends	−0.3215	0.0693	−4.64	**0.000**	−0.4574	−0.1856
Pedastr * parents	−0.5454	0.0823	−6.63	**0.000**	−0.7068	−0.3841
Countryside * bike	−0.3942	0.0615	−6.41	**0.000**	−0.5148	−0.2736
Countryside * motor tr	0.1397	0.0486	2.87	**0.004**	0.0442	0.2351
Countryside * 30 min	0.3041	0.0536	5.67	**0.000**	0.1990	0.4092
Countryside * 45 min	0.8876	0.0535	16.58	**0.000**	0.7826	0.9925
Countryside * friends	−0.5941	0.0516	−11.50	**0.000**	−0.6953	−0.4928
Countryside * parents	−0.8464	0.0605	−13.98	**0.000**	−0.9650	−0.7278
Traffic street * bike	−0.2867	0.0773	−3.71	**0.000**	−0.4382	−0.1352
Traffic street * motor tr	0.0691	0.0612	1.13	0.259	−0.0508	0.1891
Traffic street * 30 min	0.2237	0.0676	3.31	**0.001**	0.0910	0.3564
Traffic street * 45 min	0.4923	0.0668	7.36	**0.000**	0.3612	0.6234
Traffic street * friends	−0.4105	0.0652	−6.29	**0.000**	−0.5384	−0.2826
Traffic street * parents	−0.6328	0.0771	−8.21	**0.000**	−0.7839	−0.4817
Industry * bike	−0.3597	0.1731	−2.08	**0.038**	−0.6990	−0.0204
Industry * motor tr	0.04917	0.1259	0.39	0.696	−0.1977	0.2960
Industry * 30 min	−0.11419	0.1449	−0.79	0.431	−0.3983	0.1699
Industry * 45 min	0.2372	0.1447	1.64	0.101	−0.0465	0.5209
Industry * friends	−0.6263	0.1446	−4.33	**0.000**	−0.9098	−0.3428
Industry * parents	−0.5663	0.1738	−3.26	**0.001**	−0.9070	−0.2257

* indicates the interaction between two variables; Coef. = regression coefficients; Std. Err. = standard error; z = z value (test statistic z is the ratio of the Coef. to the Std. Err. of the respective predictor); *P* > |z| = probability that the z value would be observed under the null hypothesis that a particular predictor’s regression coefficient is zero, given that the rest of the predictors are in the model; 95% Conf. Interval = 95% confidence interval; the bold indicates statistical significant results; e- in the coefficients indicates an exponential coefficient.

**Table A7:** Analysis of interaction effects driving adolescents’ preferences: full model considering together (i) adolescents’ socio-demographics and personal characteristics; (ii) city size/typology where adolescents live; (iii) type of neighborhood.

Variable	Coef.	Std. Err.	z	*p* > |z|	[95% Conf. Interval]	
By bike	−0.8215	0.1362	−6.03	**0.000**	−1.0886	−0.5543
Motor transport	−0.1303	0.1044	−1.25	0.212	−0.3350	0.0742
30 min	0.3911	0.1152	3.39	**0.001**	0.1652	0.6169
45 min	1.1073	0.1154	9.59	**0.000**	0.8810	1.3336
With friends	−0.7219	0.1130	−6.39	**0.000**	−0.9434	−0.5004
With parents	−0.9290	0.1319	−7.04	**0.000**	−1.1877	−0.6703
Female * bike	0.3360	0.0760	4.42	**0.000**	0.1868	0.4851
Female * motor tr	0.0898	0.0590	1.52	0.128	−0.0257	0.2055
Female * 30 min	−0.0684	0.0654	−1.05	0.296	−0.1966	0.0597
Female * 45 min	−0.0644	0.0654	−0.99	0.325	−0.1926	0.0637
Female * friends	0.0544	0.0637	0.85	0.393	−0.07050	0.1794
Female * parents	−0.1385	0.0755	−1.83	0.067	−0.2866	0.0095
BMI * bike	1.91 × 10^−7^	1.13 × 10^−6^	0.17	0.866	−2.02 × 10^−6^	2.40 × 10^−6^
BMI * motor	1.11 × 10^−6^	6.24 × 10^−7^	1.77	0.076	−1.17 × 10^−7^	2.33 × 10^−6^
BMI * 30 min	6.71 × 10^−8^	6.71 × 10^−7^	0.10	0.920	−1.25 × 10^−6^	1.38 × 10^−6^
BMI * 45 min	−8.59 × 10^−7^	7.56 × 10^−7^	−1.14	0.256	−2.34 × 10^−6^	6.22 × 10^−7^
BMI * friends	2.34 × 10^−7^	6.74 × 10^−7^	0.35	0.728	−1.09 × 10^−6^	1.55 × 10^−6^
BMI * parents	7.47 × 10^−7^	7.55 × 10^−7^	0.99	0.322	−7.33 × 10^−7^	2.23 × 10^−6^
Low Adher * bike	−0.0559	0.1693	−0.33	0.741	−0.3879	0.2759
Low Adher * motor tr	0.6003	0.1308	4.59	**0.000**	0.3439	0.8567
Low Adher * 30 min	−0.2300	0.1447	−1.59	0.112	−0.5136	0.0535
Low Adher * 45 min	−0.1872	0.1438	−1.30	0.193	−0.4691	0.0946
Low Adher * friends	0.1211	0.1405	0.86	0.389	−0.1542	0.3966
Low Adher * parents	−0.0123	0.1662	−0.07	0.941	−0.3382	0.3135
Medium adher * bike	−0.0593	0.1208	−0.49	0.623	−0.2961	0.1774
Medium adher * motor tr	0.2511	0.0939	2.67	**0.008**	0.0670	0.4352
Medium adher * 30 min	0.0142	0.1027	0.14	0.890	−0.1871	0.2156
Medium adher * 45 min	0.0668	0.1023	0.65	0.514	−0.1337	0.2674
Medium adher * friends	−0.0402	0.1009	−0.40	0.690	−0.2380	0.1574
Medium adher * parents	−0.1651	0.1170	−1.41	0.158	−0.3945	0.0642
Assoc member * bike	0.2511	0.0779	3.22	**0.001**	0.0983	0.4039
Assoc member * motor tr	0.1909	0.0605	3.15	**0.002**	0.0722	0.3096
Assoc member * 30 min	0.0843	0.0664	1.27	0.204	−0.0458	0.2145
Assoc member * 45 min	0.0618	0.0667	0.93	0.354	−0.0689	0.1926
Assoc member * friends	−0.1445	0.0654	−2.21	**0.027**	−0.2728	−0.0162
Assoc member * parents	−0.1175	0.0769	−1.53	0.126	−0.2682	0.0331
60 min/day * bike	0.1113	0.0771	1.44	0.149	−0.0397	0.2624
60 min/day * motor tr	−0.2270	0.0604	−3.76	**0.000**	−0.3455	−0.1085
60 min/day * 30 min	0.0355	0.0664	0.53	0.593	−0.0946	0.1656
60 min/day * 45 min	−0.1535	0.0662	−2.32	**0.021**	−0.2834	−0.0235
60 min/day * friends	0.0730	0.0649	1.13	0.260	−0.0541	0.2003
60 min/day * parents	0.1703	0.0762	2.23	**0.025**	0.0208	0.3197
Metropolis * bike	0.1058	0.7980	0.13	0.894	−1.4583	1.6700
Metropolis * motor tr	−0.2638	0.5071	−0.52	0.603	−1.2579	0.7301
Metropolis * 30 min	−1.026	0.5777	−1.78	0.076	−2.1589	0.1059
Metropolis * 45 min	−0.8086	0.6152	−1.31	0.189	−2.0144	0.3972
Metropolis * friends	−0.4211	0.6044	−0.70	0.486	−1.6058	0.7635
Metropolis * parents	−0.2541	0.7424	−0.34	0.732	−1.7092	1.2009
City * bike	0.0012	0.1458	0.01	0.993	−0.2846	0.2872
City * motor tr	−0.2231	0.1122	−1.99	**0.047**	−0.4432	−0.0030
City * 30 min	0.0205	0.1212	0.17	0.866	−0.2171	0.2582
City * 45 min	0.0012	0.1238	0.01	0.992	−0.2415	0.2440
City * friends	0.1102	0.1213	0.91	0.363	−0.1275	0.3480
City * parents	−0.1570	0.1426	−1.10	0.271	−0.4366	0.1225
Town * bike	0.0162	0.0780	0.21	0.835	−0.1366	0.1691
Town * motor tr	−0.0713	0.0602	−1.18	0.236	−0.1894	0.0466
Town * 30 min	−0.0937	0.0666	−1.41	0.159	−0.2243	0.0368
Town * 45 min	−0.0388	0.0666	−0.58	0.561	−0.1695	0.0919
Town * friends	0.0158	0.0652	0.24	0.808	−0.1120	0.1438
Town * parents	0.0425	0.0769	0.55	0.580	−0.1083	0.1934
Park * bike	−0.1133	0.0748	−1.51	0.130	−0.2599	0.0333
Park * motor tr	−0.1687	0.0576	−2.93	**0.003**	−0.2817	−0.0558
Park * 30 min	−0.0683	0.0635	−1.08	0.282	−0.1929	0.0562
Park * 45 min	−0.0849	0.0637	−1.33	0.183	−0.2098	0.0400
Park * friends	−0.0025	0.0624	−0.04	0.967	−0.1250	0.1198
Park * parents	−0.0350	0.0735	−0.48	0.634	−0.1791	0.1091
Pedastr * bike	−0.0654	0.0905	−0.72	0.470	−0.2429	0.1120
Pedastr * motor tr	−0.1655	0.0690	−2.40	**0.017**	−0.3008	−0.0301
Pedastr * 30 min	−0.0140	0.0769	−0.18	0.856	−0.1648	0.1368
Pedastr * 45 min	0.0030	0.0774	0.04	0.969	−0.1488	0.1548
Pedastr * friends	0.0451	0.0750	0.60	0.547	−0.1018	0.1921
Pedastr * parents	−0.0377	0.0885	−0.43	0.670	−0.2113	0.1358
Traffic street * bike	0.0321	0.0864	0.37	0.710	−0.1372	0.2016
Traffic street * motor tr	0.0070	0.0674	0.11	0.916	−0.1250	0.1392
Traffic street * 30 min	0.0503	0.0747	0.67	0.500	−0.0960	0.1968
Traffic street * 45 min	−0.0622	0.0743	−0.84	0.403	−0.2080	0.0835
Traffic street * friends	−0.0043	0.0725	−0.06	0.952	−0.1466	0.1379
Traffic street * parents	−0.0389	0.0857	−0.45	0.650	−0.2070	0.1292
Industry * bike	−0.1423	0.1783	−0.80	0.425	−0.4919	0.2072
Industry * motor tr	−0.0034	0.1292	−0.03	0.979	−0.2568	0.2499
Industry * 30 min	−0.1758	0.1465	−1.20	0.230	−0.4631	0.1114
Industry * 45 min	−0.1091	0.1465	−0.74	0.456	−0.3963	0.1781
Industry * friends	−0.3999	0.1483	−2.70	**0.007**	−0.6907	−0.1092
Industry * parents	−0.2070	0.1751	−1.18	0.237	−0.5502	0.1361

* indicates the interaction between two variables; Coef. = regression coefficients; Std. Err. = standard error; z = z value (test statistic z is the ratio of the Coef. to the Std. Err. of the respective predictor); *P* > |z| = probability that the z value would be observed under the null hypothesis that a particular predictor’s regression coefficient is zero, given that the rest of the predictors are in the model; 95% Conf. Interval = 95% confidence interval; the bold indicates statistical significant results; e- in the coefficients indicates an exponential coefficient.
